# Construction and in vivo assembly of a catalytically proficient and hyperthermostable de novo enzyme

**DOI:** 10.1038/s41467-017-00541-4

**Published:** 2017-08-25

**Authors:** Daniel W. Watkins, Jonathan M. X. Jenkins, Katie J. Grayson, Nicola Wood, Jack W. Steventon, Kristian K. Le Vay, Matthew I. Goodwin, Anna S. Mullen, Henry J. Bailey, Matthew P. Crump, Fraser MacMillan, Adrian J. Mulholland, Gus Cameron, Richard B. Sessions, Stephen Mann, J. L. Ross Anderson

**Affiliations:** 1 School of Biochemistry, University of Bristol, University Walk, Bristol, BS8 1TD UK; 2 BrisSynBio Synthetic Biology Research Centre, Life Sciences Building, University of Bristol, Tyndall Avenue, Bristol, BS8 1TQ UK; 3 School of Chemistry, University of Bristol, Bristol, BS8 1TS UK; 4 Henry Wellcome Unit of Biological EPR, School of Chemistry, University of East Anglia, Norwich, NR4 7TJ UK

## Abstract

Although catalytic mechanisms in natural enzymes are well understood, achieving the diverse palette of reaction chemistries in re-engineered native proteins has proved challenging. Wholesale modification of natural enzymes is potentially compromised by their intrinsic complexity, which often obscures the underlying principles governing biocatalytic efficiency. The maquette approach can circumvent this complexity by combining a robust de novo designed chassis with a design process that avoids atomistic mimicry of natural proteins. Here, we apply this method to the construction of a highly efficient, promiscuous, and thermostable artificial enzyme that catalyzes a diverse array of substrate oxidations coupled to the reduction of H_2_O_2_. The maquette exhibits kinetics that match and even surpass those of certain natural peroxidases, retains its activity at elevated temperature and in the presence of organic solvents, and provides a simple platform for interrogating catalytic intermediates common to natural heme-containing enzymes.

## Abstract (web summary)

Catalytic mechanisms of enzymes are well understood, but achieving diverse reaction chemistries in re-engineered proteins can be difficult. Here the authors show a highly efficient and thermostable artificial enzyme that catalyzes a diverse array of substrate oxidations coupled to the reduction of H_2_O_2_.

## Introduction

Tailor-made protein catalysts that are compatible with the natural biomolecular components of living cells are key to realizing the ambitious goals of synthetic biology and to the provision of cheap, green catalysts for industrial biotechnology^1–3^. While there have been notable successes in designing de novo enzymes from engineered natural protein scaffolds^4–6^, the inherent complexity of evolved proteins is not a prerequisite for sustaining sophisticated functions supporting enzymatic catalysis and may even hinder the acquisition and honing of enzymatic function in such proteins^1–3^. Indeed, it has been demonstrated that simple, manmade proteins can reproduce selected functions and catalytic activities of natural metalloenzymes^7–10^. Maquettes—simple 4-α-helix bundles designed from first principles^3^—in particular provide a blank canvas for tractable and iterative design processes, as demonstrated by the successful incorporation of sophisticated functions common to oxidoreductases such as oxygen binding^11^ and intermolecular electron transfer^3^. However, no enzymatic activity has yet been integrated into a maquette. We recently demonstrated that maquettes can be recognized by, and processed through, the post-translational cytochrome *c* maturation machinery in the periplasmic space of *E. coli* to covalently graft heme onto the maquette backbone^12, 13^. These *c*-type cytochrome maquettes (CTMs) retain the functional legacy of their maquette precursors (e.g., oxygen binding), while providing a robust, functional framework for further oxidoreductase engineering. That the CTMs are fully and functionally assembled in vivo affords an opportunity to examine de novo protein function within living cells and facilitates the use of directed evolution strategies to improve incipient function.

Here, we demonstrate the power of the maquette approach and the utility of the CTMs through the construction of a catalytically proficient de novo enzyme. Our strategy is based on a simple, two-step conversion of a non-catalytic, CTM to produce a catalytically active holoprotein from *E. coli*. Remarkably, this elementary design process yields a de novo enzyme that exhibits steady-state kinetics for oxidation and oxidative dehalogenation that match and even surpass those of natural peroxidases, while providing a thermostable and chemically resistant chassis for biocatalysis.

## Results

### C45 design and biophysical characterization

**Fig. 1:**
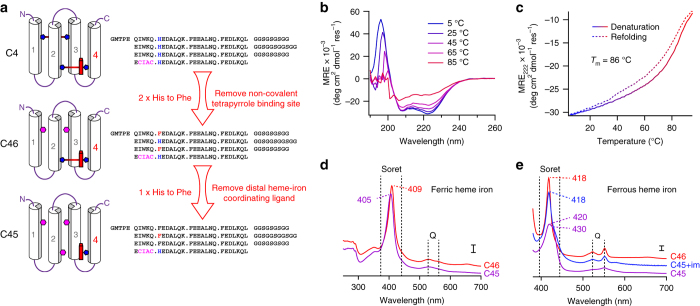
Design and characterization of an artificial peroxidase. **a** The design process begins with C4, a CTM containing a bis-histidine heme C-binding site with the consensus motif for covalent heme incorporation on helix 4 with the distal histidine heme ligand on helix 2, and a second non-covalent tetrapyrrole-binding site (occluded by helix 2) consisting of a bis-histidine pair on helices 1 and 3. Mutation of both histidines of the non-covalent-binding site to phenylalanine produces the CTM, C46. Subsequent mutation of the distal heme C-ligating histidine ligand results in the mono-histidine ligated C45. Purification data are displayed in Supplementary Fig. 1. **b** Far-UV circular dichroism spectra of C45 with varying temperature collected in 100 mM KCl, 20 mM CHES, pH 8.58. **c** Temperature dependence of the CD signal monitored at 222 nm during denaturation (*solid line*) and refolding (*dashed line*). **d**, **e** UV/visible spectra of ferric (**d**) and ferrous (**e**) C45 (*purple*) and C46 (*red*). The spectrum of ferrous C45 with added exogenous imidazole is displayed in *blue*. *Scale bars* represent optical densities of 0.1

To expand the functional repertoire of our maquettes, we selected coupled substrate oxidation/H_2_O_2_ reduction for inclusion into a CTM. The natural heme-containing peroxidases that catalyze this chemistry typically contain heme B or C axially coordinated by a single histidine side chain^14^. We hypothesized that catalytic turnover of hydrogen peroxide within a maquette scaffold could be achieved by the construction of a monohistidine-ligated CTM from an existing CTM scaffold. To this end, we first selected a CTM with heme covalently appended to helix 4 (C4) owing to its increased thermal stability over the original parent design^13^. To restrict conformational flexibility and improve hydrophobic core packing, we replaced both histidines of the second, non-covalent tetrapyrrole-binding site with phenylalanine (C46) (Fig. 1a, Supplementary Fig. 1A, C, E, G), removing the capacity to bind a second tetrapyrrole and increasing the melting transition temperature (*T*_m_) by 31 to 84 °C (Supplementary Fig. 2). Subsequent replacement of the distal histidine on helix 2 with phenylalanine yielded the monohistidine-ligated CTM C45; a predominantly helical protein that retains the hyperthermophilic characteristics of its precursor (*T*_m_(C45) = 86 °C) and reversibly refolds following thermal denaturation (Fig. 1b, c; Supplementary Fig. 1B, D, F, H).

C45 exhibits ferric and ferrous UV/visible spectra (Fig. 1d, e) similar to those of the monohistidine-ligated cytochrome *c*′^15^ and horse heart cytochrome *c* distal methionine mutants^16^, suggesting either a quantum mechanical admixture of low-spin and high-spin states exists at the heme iron^17^ or a titratable water molecule occupies the vacant axial coordination site^18, 19^. The ferric C45 spectrum is pH dependent (Supplementary Fig. 3A, B), yielding a p*K*_a_ of 8.11 for the transition between spectra at neutral and alkaline pH (pH 6.5–11), similar to the acid-alkaline transition p*K*_a_ of horse heart myoglobin (p*K*_a_ = 8.9)^18^ and indicative of a heme C-bound water molecule occupying the available axial heme ligation site. C45 displays a marked pH-dependence in its heme redox potential (Supplementary Fig. 3C–E), increasing from −208 mV at pH 10 to −174 mV at pH 8.6. At pH 7.5 two redox potentials are observed, likely reflecting a mixture of spin states and the water/hydroxide equilibrium at the distal coordination site. The exogenous diatomic ligand-binding function of its CTM precursors^12, 13^ is retained in C45: it readily binds carbon monoxide and oxygen in the ferrous form, and cyanide in the ferric form (Supplementary Fig. 4A, B). The ability to bind oxygen is strongly indicative of the retention, from previous designs, of a relatively dry and conformationally stable oxygen-binding site on the distal heme face^11, 12^. O_2_ binds 10-fold more rapidly to C45 than the bis-histidine ligated C4^13^ (Supplementary Fig. 4C, D). Imidazole readily binds to either ferric or ferrous C45, reproducing the spectra of the six-coordinate C46 and C4 CTMs (Fig. 1d, e).

### Steady-state kinetics and substrate promiscuity of C45

**Fig. 2:**
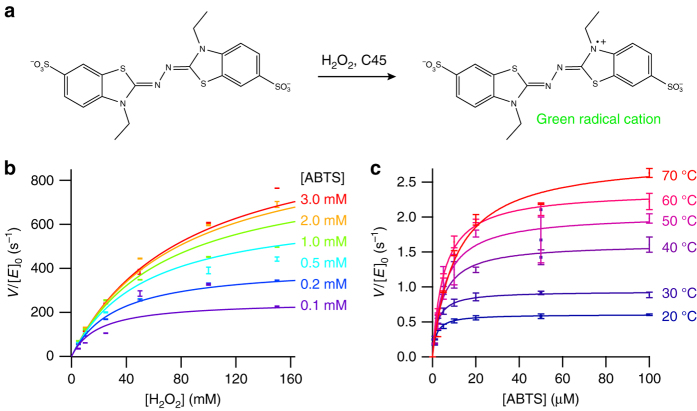
Steady-state kinetics of ABTS and hydrogen peroxide turnover by C45. **a** Transformation of ABTS to a green radical cation as catalyzed by natural peroxidases and C45. **b** Steady-state kinetics plot for ABTS turnover by C45 at varying [ABTS] and [H_2_O_2_]. Data are fit to a ping-pong steady-state kinetics model. **c** The thermal stability of C45 enables classical Michaelis–Menten kinetics to be determined up to 70 °C. All data were recorded in triplicate and *error bars* represent the standard deviation. Michaelis–Menten parameters for the data contained in **c** are presented in Supplementary Table 1. Kinetic data were collected in 20 mM CHES, 100 mM KCl, pH 8.58 with 0.1 μM C45

When rapidly mixed with hydrogen peroxide and the classical peroxidase substrate ABTS (2,2′-azino-bis(3-ethylbenzothiazoline-6-sulfonic acid))^14^, C46 very slowly bleaches indicating heme degradation with no evidence of spectroscopically isolable intermediates and little oxidized ABTS product. In contrast, when rapidly mixed with hydrogen peroxide, the monohistidine-ligated ferric C45 rapidly reacts with ABTS to form the green ABTS radical cation (Fig. 2a, Supplementary Fig. 5A). At the optimum reaction pH (pH = 8.58, Supplementary Fig. 6A, B) the reaction between C45 and H_2_O_2_/ABTS follows the ping-pong steady-state kinetics typical of natural peroxidases^20^ (Fig. 2b, Supplementary Fig. 7) (*k*_cat_ = 1200 s^−1^; *K*_m_(H_2_O_2_) = 94 mM; *K*_m_(ABTS) = 379 μM), with exceptional catalytic efficiency for the electron transfer between peroxide-activated C45 and ABTS (*k*_cat_/*K*_m_ = 3.2 × 10^6^ M^−1^ s^−1^). This catalytic efficiency is among the highest observed in a de novo enzyme and matches well that of horseradish peroxidase (HRP) operating at its optimal pH (*k*_cat_ = 4100 s^−1^; *K*_m_(ABTS) = 800 μM; *k*_cat_/*K*_m_(ABTS) = 5.13 × 10^6^ M^−1^ s^−1^)^21^. Despite the high efficiency of the electron transfer step, the activation of peroxide by C45 is less catalytically efficient than in HRP (C45 *k*_cat_/*K*_m_ = 1.3 × 10^4^ M^−1^ s^−1^; HRP *k*_cat_/*K*_m_ = 4.6 × 10^6^ M^−1^ s^−1^ at pH 7.5)^22^, and is likely due to the lack of peroxide-activating amino acid side chains in the vicinity of the heme. Compared to other de novo heme-containing proteins that exhibit peroxidase activity, C45 is markedly more catalytically efficient and does not require helix-stabilizing additives such as trifluoroethanol for catalytic activity^23–25^. C45 is remarkably resilient to both elevated temperature (Fig. 2c) and the presence of organic solvents (Supplementary Fig. 6C, D), and retains impressive catalytic efficiency close to its denaturation temperature.

**Fig. 3:**
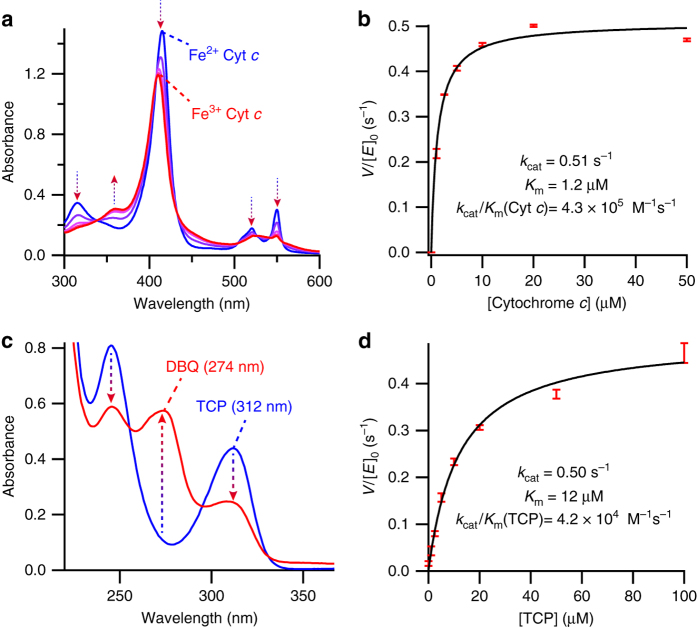
Cytochrome *c* oxidation and TCP oxidative dehalogenation catalyzed by C45. **a** Representative changes to the UV/visible spectrum of equine cytochrome *c* during C45-catalyzed oxidation. **b** Michaelis–Menten plot of equine cytochrome *c* oxidation catalyzed by C45 in the presence of limiting H_2_O_2_ (100 μM). **c** Representative changes to the UV/visible spectrum of TCP during C45-catalyzed oxidative dehalogenation to the dichloroquinone product (DBQ). **d** Michaelis–Menten plot of 2,4,6-trichlorophenol oxidative dehalogenation catalyzed by C45 in the presence of limiting H_2_O_2_ (100 μM). All data were recorded in triplicate and *error bars* represent the standard deviation

Previous work by Fry et al.^26^ has illustrated that heme maquettes can undergo rapid interprotein electron transfer with natural proteins such as cytochrome *c*, facilitated by complementary surface electrostatic interactions. Since C45 presents similar electrostatic surfaces to these maquettes, we reasoned that ferrous cytochrome *c* could act as an electron donor in the peroxidase reaction with C45, reproducing the activity of the natural cytochrome *c* peroxidases^14^. C45 is indeed capable of functioning as an efficient artificial cytochrome *c* peroxidase (Fig. 3a, b; Supplementary Fig. 5B), though the high *K*_m_(H_2_O_2_) of C45 precludes the determination of precise enzyme mechanism due to the high background rate of ferrous cytochrome *c* oxidation at millimolar H_2_O_2_ concentrations. We therefore fit the data to a simple Michaelis–Menten steady-state model with *k*_cat_ here representing the observed maximum rate constant at limiting H_2_O_2_ concentration (100 μM). C45 exhibits impressive catalytic efficiency at limiting H_2_O_2_ concentrations (*k*_cat_/*K*_m_(cyt*c*) = 4.3 × 10^5^ M^−1^ s^−1^ vs. *k*_cat_/*K*_m_(cyt*c*) = 1.62 × 10^7^ M^−1^ s^−1^ for the natural yeast cytochrome *c* peroxidase^27^); however, under non-limiting concentrations of peroxide, we predict that *k*_cat_ and *k*_cat_/*K*_m_(cyt*c*) will be significantly increased. Nevertheless, this demonstrates the ability of C45 to couple interprotein electron transfer to a chemical transformation at a protein-bound heme, an intrinsic feature of respiratory enzymes such as cytochrome oxidase^28^.

Much like the natural heme-containing peroxidases^14^, C45 exhibits significant substrate promiscuity: it catalyzes the oxidation of peroxidase substrates including guaiacol, *p*-anisidine, *o*-phenylenediamine, 5-aminosalicylic acid, luminol, reactive blue 4, reactive black 5, and the anti-tuberculosis prodrug, isoniazid (Supplementary Fig. 8). This catalytic promiscuity also extends to the oxidative dehalogenation of halogenated phenols such as 2,4,6-trichlorophenol (TCP) (Supplementary Fig. 5C) and its bromo-analog and fluoro-analog, and 4-bromophenol. Like the ferrous cytochrome *c* oxidation described above, the mechanistic interrogation of TCP dehalogenation by C45 and H_2_O_2_ is hampered by the high C45 *K*_m_(H_2_O_2_) and incompatibility of the assay with millimolar [H_2_O_2_], and the data were therefore analyzed using a simple Michaelis–Menten model (Fig. 3c, d) at a limiting hydrogen peroxide concentration (100 μM). Compared to the dehaloperoxidase from *Amphitrite ornata* ^29^, an enzyme with a globin evolutionary heritage, C45 improves 5-fold on the natural enzyme’s catalytic efficiency at this limiting H_2_O_2_ concentration (*k*_cat_/*K*_m_(TCP) = 4.2 × 10^4^ M^−1^ s^−1^ vs. *k*_cat_/*K*_m_(TCP) = 8.1 × 10^3^ M^−1^ s^−1^ for DHP A). We predict *k*_cat_ and *k*_cat_/*K*_m_(TCP) values at non-limiting H_2_O_2_ concentrations also to be significantly higher than for the natural enzyme.

### Isolation of a reactive intermediate

**Fig. 4:**
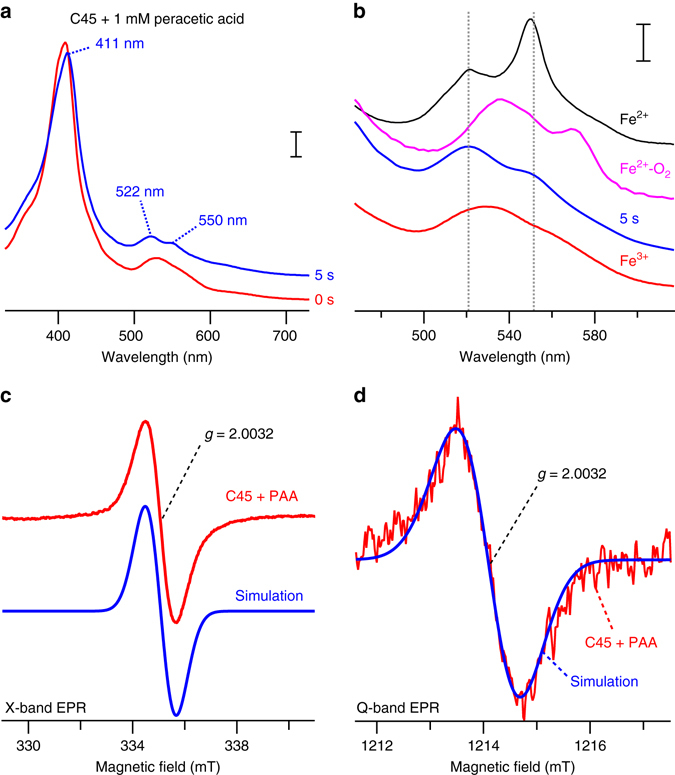
Isolation of high-valent oxo-iron species in a CTM. **a**, **b** UV/visible spectra of ferric C45 (*red*) and peracetic acid-treated C45 (*blue*) obtained by rapid mixing experiments in a stopped-flow spectrophotometer. Ferrous (*black*), Ferric (*red*), and oxyferrous C45 (*magenta*) spectra are displayed for comparison. The putative C45 compound I species in **a**, **b** were generated by mixing 2 mM peracetic acid with 20 μM ferric C45 in 100 mM KCl, 20 mM CHES, pH 8.58. *Scale bars* represent optical densities of 0.05 (**a**) and 0.02 (**b**). **c** X-band cw-EPR spectrum of C45 mixed with peracetic acid (*red*) indicates the formation of a radical species with *g* = 2.0032. Simulated data of a tryptophan radical species within C45 are presented in *blue*. Spectra were obtained by mixing 1 mM peracetic acid with C45 (700 μM) in 100 mM KCl, 20 mM CHES, pH 8.58. Experimental conditions: EPR microwave frequency = 9.3933 GHz, microwave power = 1 mW, modulation amplitude = 0.3 mT, temperature = 12 K. **d** Q-band cw-EPR spectrum of C45 mixed with peracetic acid (*red*, conditions as for the X-band EPR data) and simulated data of a tryptophan radical species in C45 are presented in *blue*. The lack of observable g-anisotropy of the radical signal indicates the presence of an amino acid side chain-based radical species. Experimental conditions: EPR microwave frequency = 34.027 GHz, microwave power = 3 μW, modulation amplitude = 0.3 mT, temperature = 50 K

To further probe the artificial peroxidase mechanism, we rapidly mixed ferric C45 with hydrogen peroxide or organic peracids in a stopped flow spectrophotometer. Approximately 5 s later, we observed a spectroscopic intermediate (Fig. 4a, b) resembling cytochrome *c* peroxidase compound I^27^. We subsequently examined this intermediate using electron paramagnetic resonance (EPR) spectroscopy and observed the appearance of a new narrow EPR signal centered around *g* = 2.0032 with a line width of 1.1 mT (peak to peak). Microwave power saturation experiments at X-band and Q-band (34 GHz) suggest that this is an isolated species indicative of an amino acid-based radical. The lack of any discernible g-anisotropy at 34 GHz indicate a tryptophan side chain as the origin of this radical species (most likely W43 which is closest to the heme), analogous to the characteristics of cytochrome *c* peroxidase compound I^30, 31^.

### NMR spectroscopy and computational modeling

**Fig. 5:**
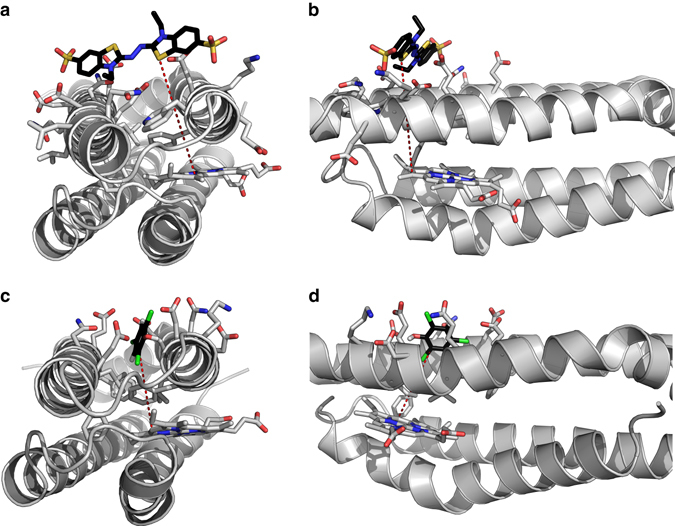
Computational scanning for substrate–C45 interactions. **a**, **b** Single snapshot from an MD simulation of lowest energy ABTS-binding site on the C45 surface derived from BUDE. **c**, **d** Single snapshot from MD simulation of lowest energy TCP-binding site on the C45 surface derived from BUDE. The carbons of the substrates are colored *black* for clarity

Despite the apparently dry interior of the protein, the 1D 1H and 2D ^1^H-^15^N SOFAST HMQC NMR spectra indicates that ferric C45 exists in a conformationally heterogeneous and dynamic state as evidenced by relatively low-peak dispersion (Supplementary Fig. 9B). However, C45 forms a discrete, monomeric, and thermally stable structure with scant evidence of conformational heterogeneity during oxygen binding. These structural characteristics and rather unusual ^1^H-^15^N spectra are observed in many heme-containing maquettes^3, 12^ and de novo proteins^32^, and evidently do not inhibit efficient peroxidase activity in C45. Indeed, there are notable examples of natural and engineered enzymes that exhibit analogous flexibility either globally or locally at the active site^33–35^. In these cases, the substrate is thought to confer structural rigidity through the induced-fit mechanism, though to date we have no experimental evidence of this behavior in C45. Though conventional substrate-binding sites are observed in some natural peroxidases, such as ascorbate peroxidase^36^, there are peroxidases for which the precise substrate:protein interactions are not definitively known^14^ and others where substrate is proven to associate at the enzyme surface rather than a well-defined cavity within the protein (e.g., lignin peroxidase)^37^. To investigate the structure and substrate interactions of C45, we constructed a computational model of C45 using Chimera^38^ and ran multiple 1 μs molecular dynamics (MD) simulations using GROMACS^39^ with the CHARMM27 forcefield^40^. C45 converges to a stable structure after 40 ns of simulation (Supplementary Fig. 10A), adopting a non-coiled-coil 4-helix bundle consistent with simulations of previous CTMs^13^ (Supplementary Fig. 10B, C). We then selected 10 snapshots of the 1 μs C45 MD simulations to capture conformational heterogeneity at the amino acid side-chain level. These 10 snapshots were then probed for ABTS and TCP binding using BUDE (Bristol University Docking Engine)^41^ in surface scanning mode, running a further 10 ns of MD simulation on the top 30 BUDE-derived complexes with highest binding energies. While the calculations indicate that ABTS may bind in up to 10 sites on the surface of the protein with similar binding energies (Fig. 5a, b; Supplementary Fig. 11A–C), there are relatively few hotspots for TCP binding despite the variation in side chain conformation in the starting C45 poses (Supplementary Fig. 11D–G). Furthermore, the highest calculated binding energy for TCP is for a cleft close to the heme (<10 Å) (Fig. 5c, d), ideal for the rapid electron tunneling necessary to support the observed catalytic rate^42^. This binding mode is stable in MD simulations. The two computational methods concur in identifying the binding site for TCP, suggestive of binding with good affinity and the indications are that this substrate-binding site is relevant for catalytic activity, offering potential for enhancing specify through designed modification. Despite the differences between the ABTS and TCP-binding interactions with C45, the substrate binding hotspots are all found at the protein surface. We therefore surmise that C45 behaves in a similar manner to lignin peroxidase^37^ and other natural peroxidases, presenting an interaction surface for substrate to dock prior to rapid electron transfer to the reactive heme intermediates.

## Discussion

Our simple, systematic construction of a functional de novo enzyme highlights the power and utility of the maquette approach to functional oxidoreductase design. Similar to the construction of the original oxygen-binding maquette^11^, the engineering steps required to attain the desired catalytic function in this simple protein scaffold are few in number, reflecting the relative ease with which evolutionarily naive proteins can be rationally engineered in a tractable, iterative process. In this case, the concomitant restriction of conformational flexibility and removal of a heme ligand were sufficient to attain efficient, multistep catalysis within a heat-resistant and solvent-resistant maquette chassis, representing just three amino acid substitutions from the oxygen-binding progenitor. Despite the relatively low-catalytic efficiency of hydrogen peroxide turnover (10^4^ M^−1^ s^−1^), the enzymatic rate constant *k*_cat_(ABTS) is among the highest yet observed in a de novo enzyme, and *k*_cat_/*K*_m_(ABTS) is greater than the catalytic efficiencies of the best reported de novo enzymes^5, 8^. Given the lack of a highly specific substrate-binding site, it is perhaps unsurprising that C45 exhibits broad substrate promiscuity. However, this catalytic promiscuity may be a general feature of primitive, nascent enzymes^43^. Since C45 is fully assembled in vivo, there is now a tantalizing opportunity to employ both rational protein design and directed evolution methodologies to optimize the catalytic chassis toward a selected substrate or chemical mechanism. We therefore anticipate that the descendants of this de novo designed enzyme will act as cheap, green, and catalytically proficient catalysts for industrial biotechnology and versatile bioblocks for synthetic biology, while providing insight into the rules underpinning the engineering of natural and designed enzymes.

## Methods

### General

All chemicals were purchased from either Sigma or Fisher Scientific, and T7 Express competent cells and Q5 polymerase were purchased from NEB.

### Molecular biology, protein expression, and purification

Mutagenesis was performed using the ligase-independent cloning/mutagenesis method of Tillett^44^. Artificial CTMs were purified from *E*. c*oli* using established procedures^12, 13^. To inhibit proteolysis, 1 mM phenylmethanesulfonyl fluoride was added to the resuspended cell pellets. These cell pastes were then lysed by sonication (Soniprep plus MSE) and the soluble lysate was collected by centrifugation (40,000 × *g* for 30 min). This was then filtered (0.22 μM syringe filter, Millipore) and applied to a 5 ml HisTrap HP IMAC column (GE Healthcare) equilibrated with 40 mM imidazole, 300 mM NaCl, 50 mM Potassium Phosphate at pH 8.0 (lysis buffer). The bound protein was then washed with lysis buffer (50 ml) and eluted with a linear gradient (0–100%) of 250 mM imidazole, 300 mM NaCl, 50 mM Potassium Phosphate at pH 8.0 (elution buffer). SDS PAGE gel analysis revealed the cleanest fractions from the column, which were subsequently pooled and dialysed for 16 h against 5 liters of 0.5 mM EDTA and 20 mM Tris pH 8.0 in a 3 kDa semi-permeable dialysis membrane. 1 mM tris(2-carboxyethyl)phosphine (TC﻿EP) or dithiothreitol﻿ (D﻿TT) was then added to the dialysed protein and TEV protease (1 μM) was added under anaerobic conditions (Belle Technology Glove Box, <5 p.p.m. O_2_) to facilitate cleavage of the N-terminal hexahistidine tag. Cleavage was complete after 5 h, and the protein was centrifuged (4000 × *g*, 10 min) and filtered (0.22 μM syringe filter, Millipore) to remove precipitated TEV protease. The protein was then loaded onto a HisTrap IMAC column equilibrated with lysis buffer as described above. Protein lacking the N-terminal hexahistidine tag eluted in the column flow through and was then placed in centrifugal concentrators (10 kDa Vivaspin Sartorius Stedim) to sufficiently increase concentration for the subsequent purification steps.

If HPLC-purification was required, the following procedure was followed using a Varian SD HPLC system: a C-18 reversed-phase HPLC column (Phenomenex) was equilibrated with 30% acetonitrile: 70% water (both with 0.1% trifluoroacetic acid). Samples (0.5 ml) were then loaded on the column at 5 ml/min and following application of 25 ml of 30% acetonitrile, a linear gradient of 30–55% acetonitrile was applied. Absorbance was monitored at 280 and 395 nm to enable assessment of protein and heme content of the eluted fractions. Fractions containing the highest heme:protein ratio from a single peak were pooled and lyophilized to remove acetronitrile and trifluoroacetic acid. Proteins were then resuspended in appropriate buffer as stated in the respective methods.

Alternatively, it was found that size-exclusion chromatography was sufficient to produce clean CTM samples. Therefore, if HPLC was not employed, then the following procedure was followed: CTM samples were concentrated to approximately 1.5 ml with a centrifugal concentrator (10 kDa Vivaspin Sartorius Stedim) and split into three 0.5 ml samples that were loaded in separate runs onto a HiLoad Superdex 75 PG column (GE Healthcare) equilibrated in 100 mM KCl, 20 mM CHES, pH 8.58, and flowing at 1 ml/min. Samples eluted in two distinct peaks, the first was aggregated protein and the second was pure maquette. The maquette peak was collected, concentrated to approximately 300 μM, and stored at 4 °C for further analysis.

### Electronic spectroscopy and stopped-flow spectrophotometry

All UV-visible spectra were recorded on an Agilent Cary-60 UV-visible spectrophotometer. CTM samples were reduced by the addition of a few grains of sodium dithionite. To confirm the presence of *c*-type heme and to enable accurate determination of heme extinction coefficients, the pyridine hemochrome method of Berry et al.^45^ was employed. Using the pyridine hemichrome and hemochrome *ε*_550 nm_ (32,700 and 8430 M^−1^ cm^−1^), extinction coefficients of *ε*_406 nm_ = 147,﻿300 M^−1^ cm^−1^ and *ε*_420 nm_ = 119,900 M^−1^ cm^−1^ were determined for the ferric and ferrous C45 Soret absorption bands.

To examine the pH-dependence of the ferric UV/visible spectrum of C45, 5 μM samples of C45 were prepared at pHs between pH 6.5 and 11.0. For pHs 6.0–6.5, 7.0–8.25, 8.5–10, and 10.5–11.0, the buffers employed were Bis-Tris, Tris, CHES, and CAPS, respectively, all at 50 mM. UV/visible spectra of the samples were measured and the Soret λ_max_ was subsequently plotted vs. pH and fitted to a double ionization model yielding two p*K*_a_ values.

Oxygen-binding kinetics of C45 were determined using a SX20 Stopped Flow Spectrophotometer (Applied Photophysics) housed in an anaerobic glove box under N_2_ ([O_2_] <5 p.p.m.; Belle Technology). C45 samples (5 μM) in 100 mM KCl, 20 mM CHES, pH 8.58 were passively degassed in the anaerobic box overnight and then reduced with a stoichiometric quantity of sodium dithionite. Using the stopped flow spectrophotometer, ferrous C45 was then rapidly mixed with oxygenated buffer (540 μM O_2_ in 100 mM KCl, 20 mM CHES, pH 8.58) at 15 °C. Oxygen concentration was determined using a Lutron PDO-520 oxygen probe. Full spectra were collected over 1 or 100 s using a diode array detector, and a single wavelength corresponding to oxyferrous C45 (569 nm) was plotted against time. This trace was then fit to two consecutive single exponential functions representing the formation of the oxyferrous complex (0–0.04 s) and its subsequent autoxidation (0.04–100 s).

Possible reactive intermediates in C45 catalysis were generated by rapidly mixing 2 mM H_2_O_2_ or 2 mM peracetic acid with 20 μM ferric C45 (in 100 mM KCl, 20 mM CHES, pH 8.58) using an SX20 Stopped Flow Spectrophotometer (Applied Photophysics) at 5 °C. Full UV/visible spectra were collected for at least 10 s after mixing.

### Circular dichroism spectroscopy

A JASCO J-815 CD polarimeter was used to measure circular dichroism spectra. Samples were typically loaded at concentrations of 0.015–0.15 mg/ml (1–10 μM) in 100 mM KCl, 20 mM CHES, pH 8.58 into a 1 mm pathlength quartz cell. Far-UV CD spectra were recorded at 100 nm/min with a sensitivity of 50 mdeg. To assess the thermal stability of the proteins, a ramp rate of 40 °C/h with 1 °C intervals was used and the ellipticity at 222 nm was measured throughout. The raw data were converted to mean residue ellipticity using the protein concentration and cell pathlength. The thermal denaturation midpoints (*T*_m_s) were assessed by plotting the second derivatives of the denaturation traces, the *x*-axis intercept corresponding to the *T*_m_.

### Nuclear magnetic resonance spectroscopy

Solutions of 0.5 mM CTM were prepared in redox buffer containing 10% v/v D_2_O (Sigma Aldrich, UK). 1D and 2D ^1^H-^15^N SOFAST HMQC spectra^46^ were acquired using a Shigemi tube (Sigma Aldrich, UK) on 30 μl of this solution using a 700 MHz Bruker AVANCE HD NMR spectrometer equipped with a 1.7 mm microcryo-coil triple resonance probe. ^15^N-enriched C45 was prepared by adding 1 g of ^15^NH_4_Cl (Cambridge Isotopes) to 1 liter of LB culture medium at the point of induction with isopropyl β-D-1-thiogalactopyran﻿oside (IPTG), following the procedure described above. It should be noted that we were unable to obtain holoprotein with heme C covalently attached when attempting to isotopically label C45 in minimal medium. Demetallated C45 was obtained using the HF:pyridine method as described previously^12^.

### EPR spectroscopy

Hydrogen peroxide or peracetic acid (1 mM) was added to C45 (700 μM in 100 mM KCl, 20 mM CHES, pH 8.58) in suprasil quartz sample tubes and flash frozen. X-band cw-EPR spectra were recorded on a Bruker eleXsys E500 spectrometer using a standard rectangular Bruker EPR cavity (ER4102T) equipped with an Oxford helium cryostat (ESR900). Experimental parameters: microwave power, 1 mW; field modulation amplitude, 3 G; field modulation frequency, 100 kHz; measuring time 160 s; temperature 12 K. Q-band cw-EPR spectra were performed on a Bruker eleXsys E-560 spectrometer using a ER 5106QT-W1 resonator equipped with a home-built ARS cryogen-free cryostat. The measured spectra were corrected for an offset against a known *g* standard [1,1-diphenyl-2-picrylhydrazyl, *g* = 2.00351 ± 0.00002]. Spectral simulations were performed using the Matlab-based Easyspin package^47^.

### Redox potentiometry

OTTLE potentiometry^48^ was used to measure heme redox potentials. CTMs (50–100 μM) were exchanged into 500 mM KCl, 10% glycerol, 100 mM KCl, 20 mM CHES, pH 8.58 and redox mediators (20 μM benzyl viologen, 20 μM anthroquinone-2-sulfonate, 20 μM phenazine, 25 μM 2-hydroxy-1,4-napthoquinone, 6 μM indigotrisulfonate, and 50 μM duroquinone) were added to facilitate equilibration between the electrodes and the redox cofactor. A home-built OTTLE cell was used for the measurements as previously described^13, 48^, consisting of a modified flat, quartz EPR tube (Wilmad, USA) with a platinum gauze working electrode, platinum counter electrode and a Ag/AgCl reference electrode (BASi, USA). A Biologic SP-150 potentiostat was used to apply potentials across the cell (+50 to −350 mV vs. NHE) in both reductive and oxidative directions to confirm equilibration, as evidenced by the lack of hysteresis in the data. To determine the heme reduction potentials, the wavelength corresponding to the heme Soret λ_max_ was plotted against the applied potential and the data were subsequently fitted to functions corresponding to a single electron (Eq. (1)) or 2 × single electron (Eq. (2)) Nernst functions

1 $${{f}}\left( {{x}} \right) = \left( {{{A}} + {{B}} \, \times  {10}^{\left( {\left( {{{E}}_{\rm{m}} - {{x}}} \right)/59} \right)}} \right)/\left( 1 + {10}^{\left( {\left( {{{E}}_{\rm{m}} - {{x}}} \right)/59} \right)} \right)$$

2 $$	f (x)= \left(A \, \times ({10} ^{((x - E_{\rm{m}} 1)/59)}) + B + C \, \times ({10} ^{((E_{\rm{m}} 2 - x) /59)})\right) \\ 	\qquad \qquad /  \left(1 + {10} ^{((x - E_{\rm{m}} 1 ) /59)} + {10} ^{((E_{\rm{m}} 2 - x) / 59)} \right)$$

In Eq. (1), *A* and *B* are *y*-axis values at 100% oxidized and reduced heme respectively; *E*_m_ is the heme reduction potential. In Eq. (2), *A* and *C* are *y*-axis values at 100% oxidized and reduced heme, respectively, and *B* represents the point on the *y*-axis where there is a changeover between absorbance contributions from the first and second electron processes; *E*_m_1 and *E*_m_2 are the heme reduction potentials.

### Steady-state kinetics

Steady-state kinetics of ABTS oxidation by C45 and H_2_O_2_ (where [ABTS] was varied) were carried out either in a 1 ml cuvette using an Agilent Cary-60 UV-visible spectrometer or on 96-well plate format (total volume per well = 300 μl) using a CLARIOstar plate reader (BMG Labtech). Unless specified, all enzymatic assays were carried out at 25 °C; enzymatic assays at elevated temperature were carried out in 1 ml cuvettes using an Agilent Cary-60 UV/visible spectrometer equipped with a TC 1 Peltier and BATH 10 circulator (Quantum Northwest). For assays conducted using the CLARIOstar plate reader, solutions of C45 and ABTS were prepared in a Sterilin 96-well plate (Thermo Fisher) at varying [ABTS] using a robotic liquid handling platform (Tecan). In all cases, reactions were initiated by the addition of H_2_O_2_ and the formation of the ABTS radical cation was monitored at 405 nm (*ε*_405 nm_ = 36,800 M^−1^ cm^−1^)^49^. Steady-state kinetics of H_2_O_2_ reduction by C45 and ABTS (where [H_2_O_2_] was varied) were carried out using a SX20 (Applied Photophysics) or HI-TECH SF-61DX2 Stopped Flow Spectrophotometer as the reaction rates were too rapid to enable measurement on a plate reader or in the standard 1 ml cuvette methods described above. 0.1 μM C45 and 24 mM ABTS (in 20 mM CHES, 100 mM KCl, pH 8.58) were rapidly mixed with varying concentrations of H_2_O_2_ between 0 and 1 M. The formation of the ABTS radical cation was followed at 525 nm (*ε*_525 nm_ = 4250 M^−1^ cm^−1^). Data were recorded in triplicate and fit to a ping pong steady-state model (Eq. (3)) using the Dynafit software package^50^.

3 $$	\nu = {[ E ]_0}{k_{\rm cat}} \! \cdot \! ((( {{{[ A ]}_0}/{K_{{\rm m}A}}} ) \! \cdot \! ( {{{[ B ]}_0}/{K_{{\rm m}B}}} ))/\\ 	\quad ( {( {{{[ A ]}_0}/{K_{{\rm m}A}}} ) + ( {{{[ B ]}_0}/{K_{{\rm m}B}}} ) + ( {{{[ A ]}_0}/{K_{{\rm m}A}}} ).( {{{[ B ]}_0}/{K_{{\rm m}B}}} )} ) )$$

To determine the pH optimum and corresponding p*K*_a_’s, ABTS oxidation kinetics were obtained at pH 6, 7, 8 (20 mM Potassium Phosphate), 8.5, 9, 10 (20 mM CHES), and 11 (20 mM CAPS). Data were fit to a double ionization model as described above.

Ferrocytochrome *c* was produced by reducing horse heart cytochrome *c* with sodium dithionite. Excess dithionite was removed using a PD-10 desalting column (GE Healthcare) and the concentration of ferrocytochrome *c* was determined using *ε*_550 nm_ = 29,500 M^−1^ cm^−1^. The reaction kinetics were determined pH 8.58 by monitoring the disappearance of the cytochrome alpha band at 550 nm and data were fit to a simple Michaelis–Menten steady-state model.

A stock solution of TCP was prepared by dissolving 2 mg of TCP in 100% ethanol and then the required TCP concentrations were obtained through serial dilution. TCP concentrations were determined using the extinction coefficient at 312 nm (*ε*_312 nm_ = 5290 M^−1^ cm^−1^)^51^. Steady-state kinetics were recorded at pH 8.58 as described above for ABTS. The reaction kinetics were determined by monitoring the formation of the dichloroquinone product (*ε*_272 nm_ (of DCQ—TCP) = 11,670 M^−1^ cm^−1^) and the kinetic data were fit to a simple Michaelis–Menten steady-state model.

To determine the effects of organic solvents, ABTS oxidation and TCP oxidative dehalogenation were carried out in the presence of increasing concentrations of ethanol and methanol, respectively. Data were collected and analyzed as described above.

### Computational methods

MD simulations of C45 were performed using GROMACS 4.6.4 (www.gromacs.org) running on local workstations and the University of Bristol HPC BlueCrystal. Gromacs tools were used to add hydrogen atoms consistent with pH 7, set up a cubic periodic box 0.18 nm large than the largest dimension of the protein and solvate the box with TIP3P water and 150 mM sodium chloride ions, ensuring overall charge neutrality. The CHARMM27 force field was modified to include heme C parameters, derived from the standard CHARMM heme B parameters^52^. An initial energy minimization was performed using steepest descents until the maximum force on any atom was less than 200 kJ mol^−1^ nm^−1^. Long-range electrostatics were treated with particle mesh Ew﻿ald (PME)﻿ and simulations performed as isothermal–isobaric ensem﻿ble (NPT) ensembles at 300 K and 1 bar maintained with v-rescale temperature coupling and isotropic Berendsen pressure coupling. An equilibration simulation was performed for 200 ps while restraining the protein atoms to their original positions. After removing restraints, the simulation was continued for 100 ns saving frames every 1 ns. Analysis and visualization was performed with the Gromacs tools and Visual Molecular Dynamics (VMD).

BUDE surface scanning mode was used for the C45 substrates ABTS and TCP on 10 different snapshots from a Gromacs MD simulation of C45. The snapshots were taken every 10 ns during the 100 ns simulation. The structures of the ligands were obtained from the ZINC database online and parameterized with the program acpype.py using Antechamber and the General Amber Force Field^53–55^. The BUDE setup, BUDE running, processing for MD, MD setup, and MD post-processing was scripted where appropriate. Each combination of ligand and snapshot of C45 simulation (receptor) had 1200 randomly assigned surface positions explored. At each surface position the ligand was docked using the BUDE EMC method (a cut-down GA) whereby 3000 initial poses were developed for a further 10 generations, expanding the 30 best parents to 3000 children each generation. In total 1.08 × 10^10^ conformations were sampled per ligand with C45. The top 50 binding modes per ligand were then processed and run as a 10 ns Gromacs simulations as described above.

### Data availability

All data are available from the authors upon reasonable request.

## Electronic supplementary material

Supplementary Information
