# Supplementary material for: Construction and in vivo assembly of a catalytically proficient and hyperthermostable de novo enzyme
Source: Nat Commun. 2017 Aug 25;8:358. doi: 10.1038/s41467-017-00541-4 (PMC5572459; doi:10.1038/s41467-017-00541-4)
Supplement: Supplementary file 1 — Supplementary Information [file 41467_2017_541_MOESM1_ESM.pdf]

Description of Supplementary Files

File Name: Supplementary Information
Description: Supplementary Figures and Supplementary Table

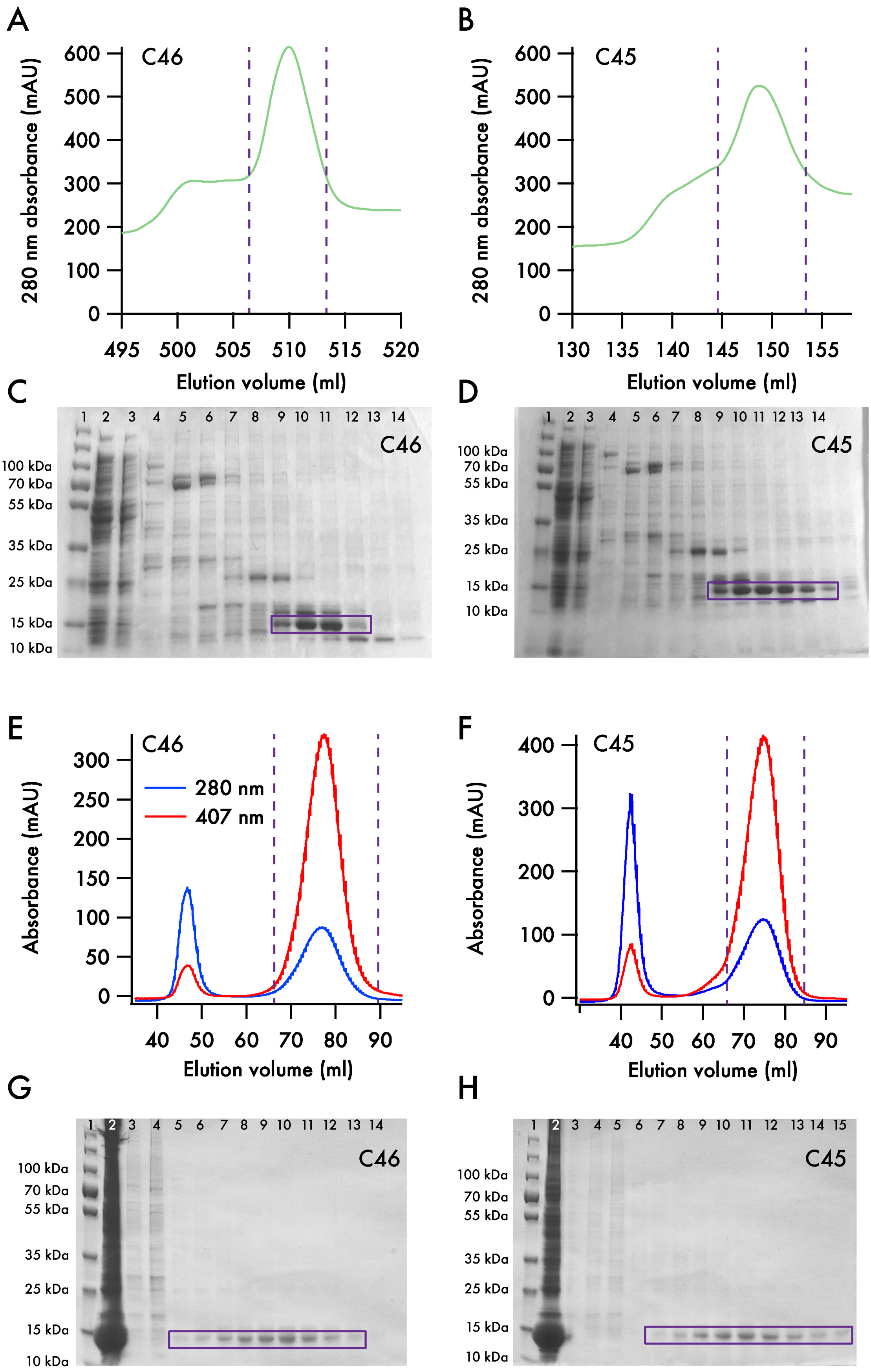


**Supplementary Figure 1. Purification of C46 and C45**

After loading crude lysate onto a nickel affinity column and washing at 40 mM imidazole, maquettes were eluted with a gradient of 40-250 mM imidazole over 15 minutes with a flow rate of 2 ml/min **(A, B)**. Fractions from the purification were analysed by SDS-PAGE gel electrophoresis **(C,D)**. Lanes

are as follows: 1. molecular weight standards. 2. sample prior to loading. 3. column flow through. 4. – 15. fractions collected in the 40 - 250 mM gradient. Fractions between the purple dashed lines were pooled for further purification. Following $His_6$-tag cleavage by TEV protease and a second nickel affinity column, maquettes were then purified by size exclusion chromatography **(E, F)**. Protein (280 nm, blue traces) and heme (407 nm, red traces) absorbances were monitored. The first peak corresponds to apo-CTMs, aggregated proteins and high molecular weight contaminants, and the second to pure holo-CTM. Fractions were subsequently analysed by SDS-PAGE gel electrophoresis **(G,H)**. Lanes are as follows: 1. molecular weight standards. 2. crude CTMs prior to loading. 3. – 14. fractions collected from the S75 column. Fractions within the purple boxes were pooled for further analysis.

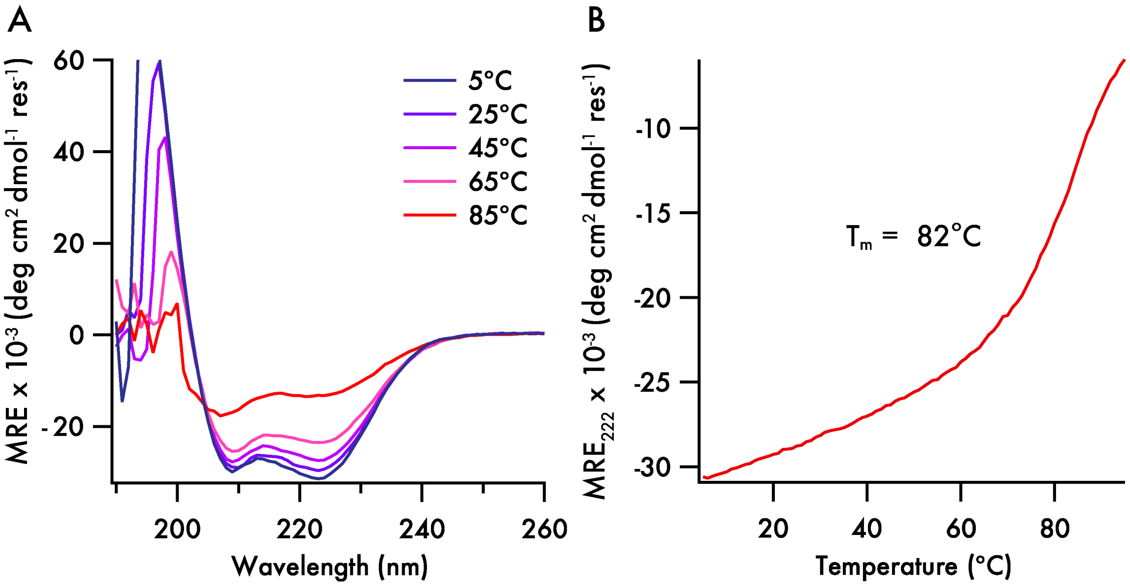


**Supplementary Figure 2. Circular dichroism spectroscopy of C46**
**A.** Far-UV circular dichroism spectra of 10 µM C46 in 100 mM KCl, 20 mM CHES, pH 8.58 collected at 5, 25, 45, 65 and 85 $^{o}$C. **B.** Mean residue ellipticity of C46 at 222 nm as a function of temperature during thermal denaturation.

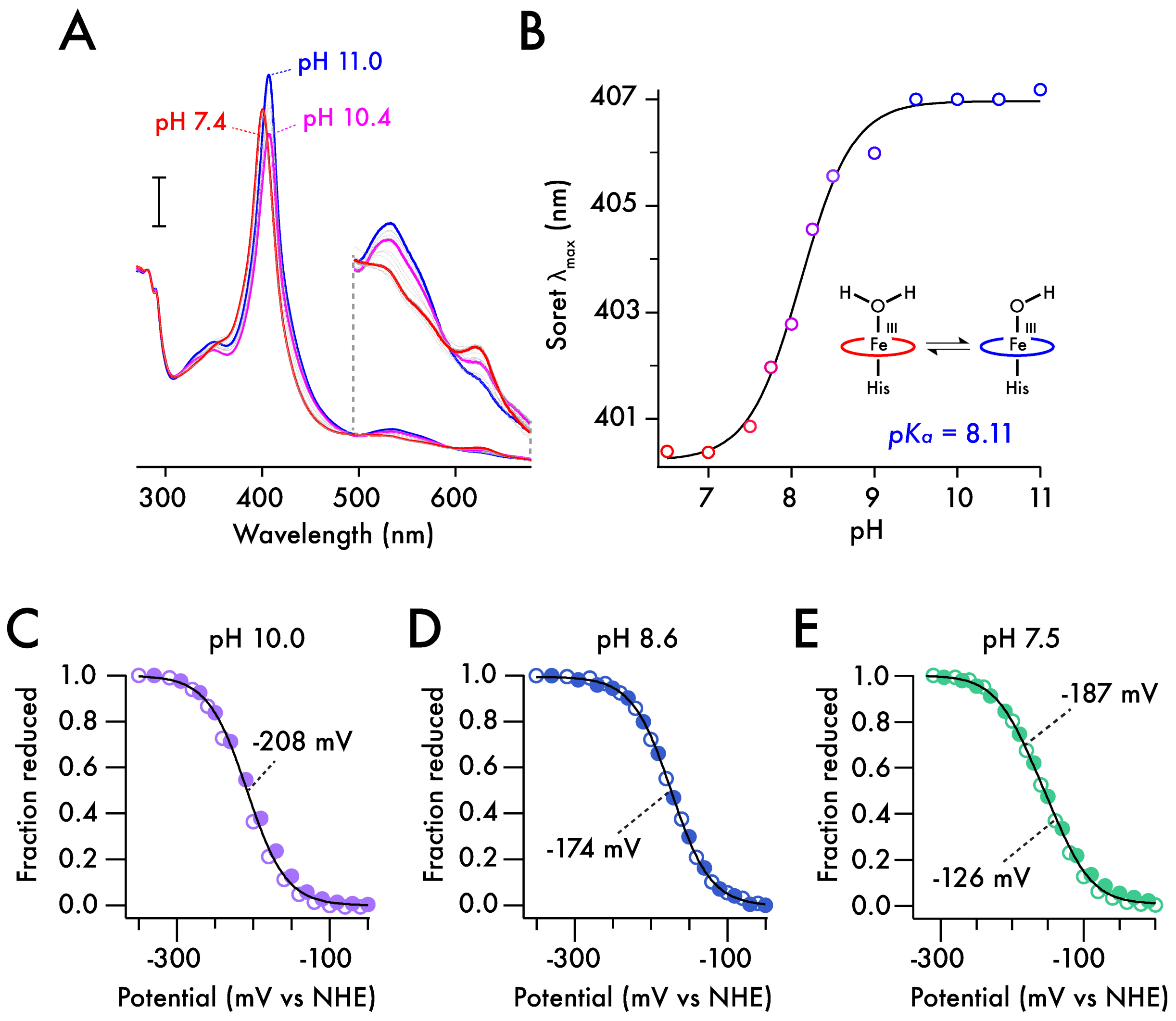


**Supplementary Figure 3. pH dependence of the Ferric C45 UV/visible spectrum and redox potential**

**A.** The UV/visible spectra of Ferric C45 recorded at pH 7.4, 10.4 and 11.0. The Soret and Q bands exhibit pH-dependent changes in intensity and $\lambda_{max}$. Scale bar represents an opical density of 0.25. **B.** Soret $\lambda_{max}$ plotted vs pH and fitted to a sigmoidal model, revealing a p$K_a$ of 8.11 for the heme-bound water/hydroxide equilibrium. **C, D, E.** Spectroelectrochemical measurements were performed in 500 mM KCl and 50 mM Buffer (CHES for pH 8.6 and 10.0; potassium phosphate for pH 7.5) at pHs 10.0 (**C**), 8.6 (**D**) and 7.5 (**E**). UV visible spectra were recorded during stepwise reduction and oxidation of the spectroelectrochemical cell. CTM absorbance at 418 nm was converted to represent the fraction of reduced protein and plotted vs applied potential. Filled circles indicate data collected during the reductive steps, open circles indicate data collected during oxidative steps. Data were fitted either to a single electron Nernst model (**C, D**) or two single electron Nernst models (**E**). Midpoint potentials are quoted vs the Nernst Hydrogen Electrode (NHE).

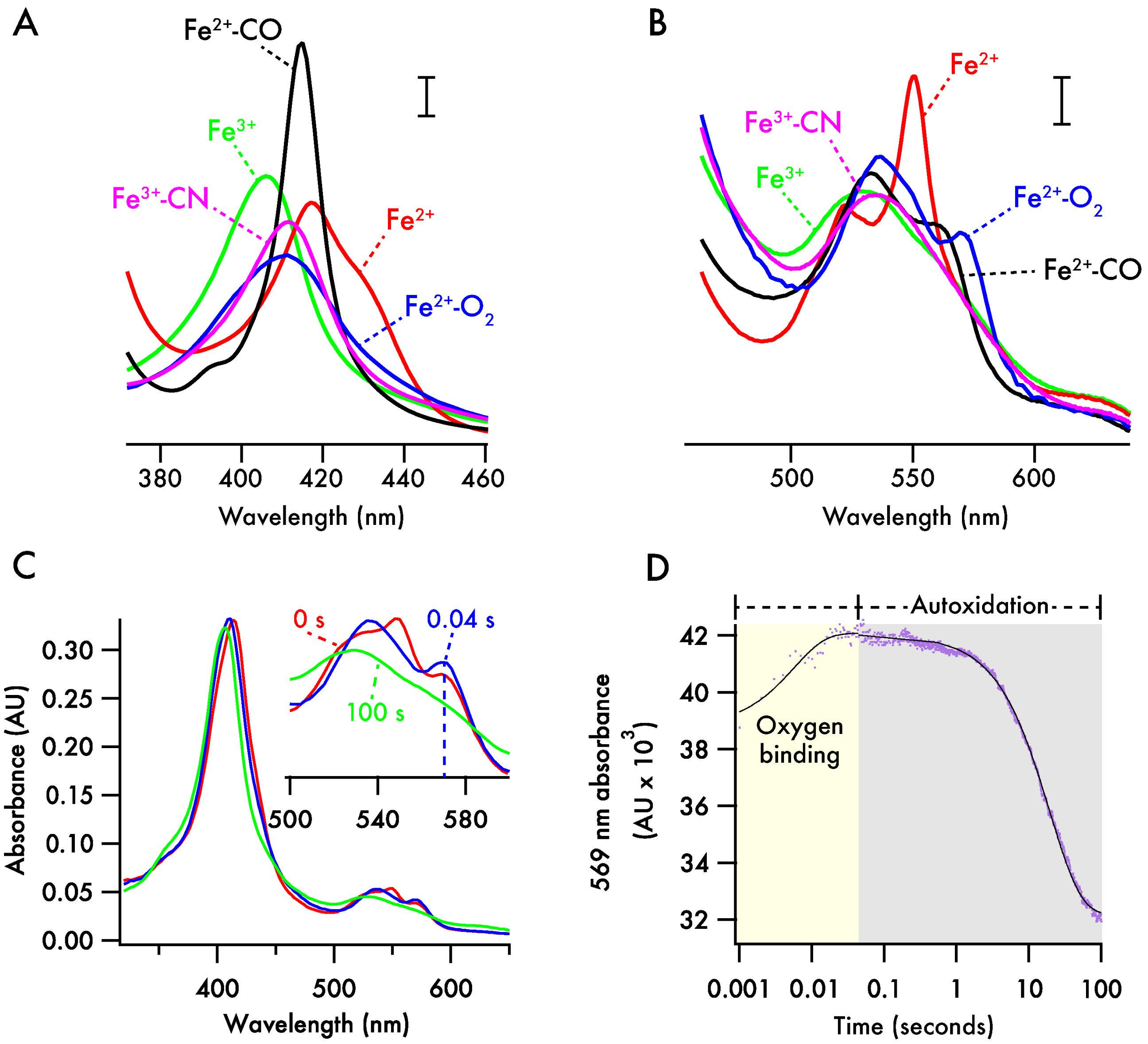


**Supplementary Figure 4. Ligand binding by ferric and ferrous C45**

**A, B.** UV/visible spectra of ferric (green), ferrous (red), oxyferrous (blue), carboxyferrous (black) and cyanoferric (magenta) C45 in the Soret band **(A)** and Q-band **(B)** regions. Spectra were recorded with 5 µM C45 in 100 mM KCl, 20 mM CHES, pH 8.58. Scale bars represent optical densities of 0.1 (**A**) and 0.01 (**B**). **C, D.** Oxygen binding to ferrous C45 measured using a stopped flow spectrophotometer (Applied Photophysics). 5 µM ferrous C45 was mixed with 540 µM $O_2$, both in 100 mM KCl, 20 mM CHES, pH 8.58. **C.** UV/visible spectra of mixed ferrous/oxyferrous (red), oxyferrous (blue) and ferric (green) C45 recorded at 0, 0.04 and 100 seconds respectively. The spectra of the Q-band region are enlarged in the inset. At this [$O_2$], oxygen binds rapidly, beginning within the dead time of the stopped flow spectrophotometer as indicated by the mixed ferrous/oxyferrous spectra at 0 seconds. **D.** Kinetics of formation and decay of the oxyferrous C45 complex. The formation and decay of the oxyferrous complex was measured at 569 nm and plotted against $\log_{10}$time (purple dots). Data were fitted to single exponential models (black traces) for the formation (0 - 0.04 seconds) and subsequent autoxidation (0.04 - 100 seconds) of the oxyferrous complex.

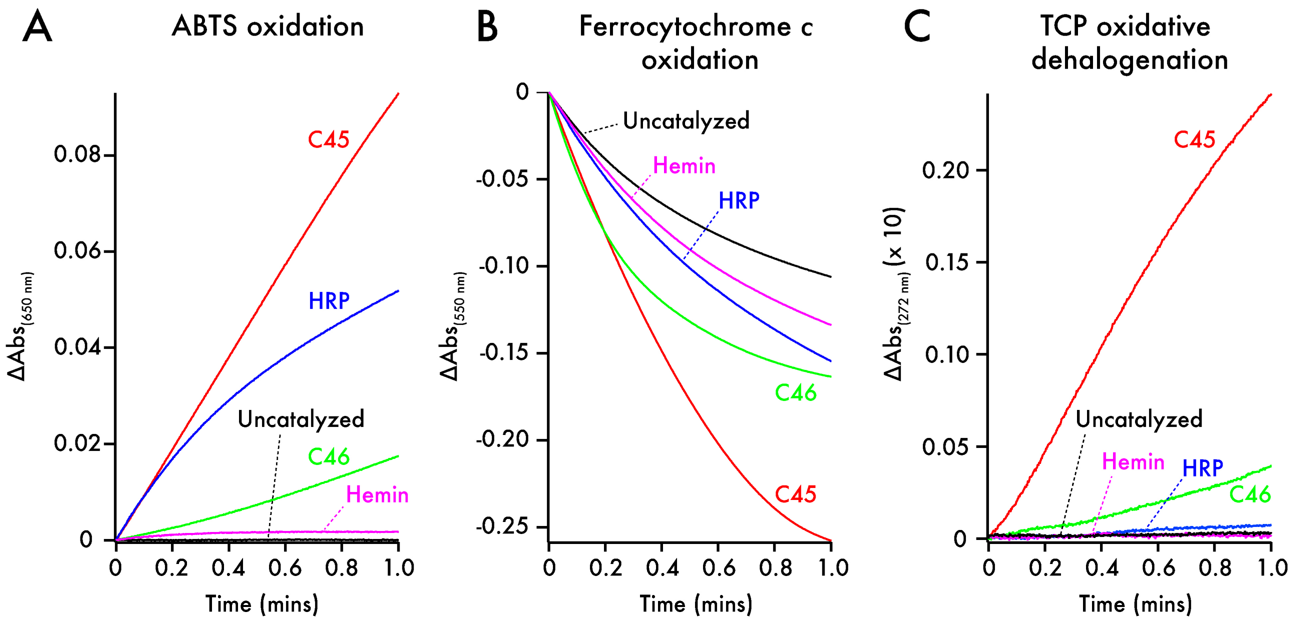


**Supplementary Figure 5. Comparison of C45 catalytic activity with C4, horseradish peroxidase and hemin**

ABTS oxidation (**A**), ferrocytochrome *c* oxidation (**B**) and TCP oxidative dehalogenation (**C**) were monitored at 650 nm, 550 nm and 272 nm respectively. In each case, equivalent concentrations of enzyme or hemin (250 nM) were added to 20 µM substrate and 100 µM $H_2O_2$ in 20 mM CHES, 100 mM KCl at pH 8.6 and 25 $^oC$. The uncatalysed background trace for each reaction is shown in black.

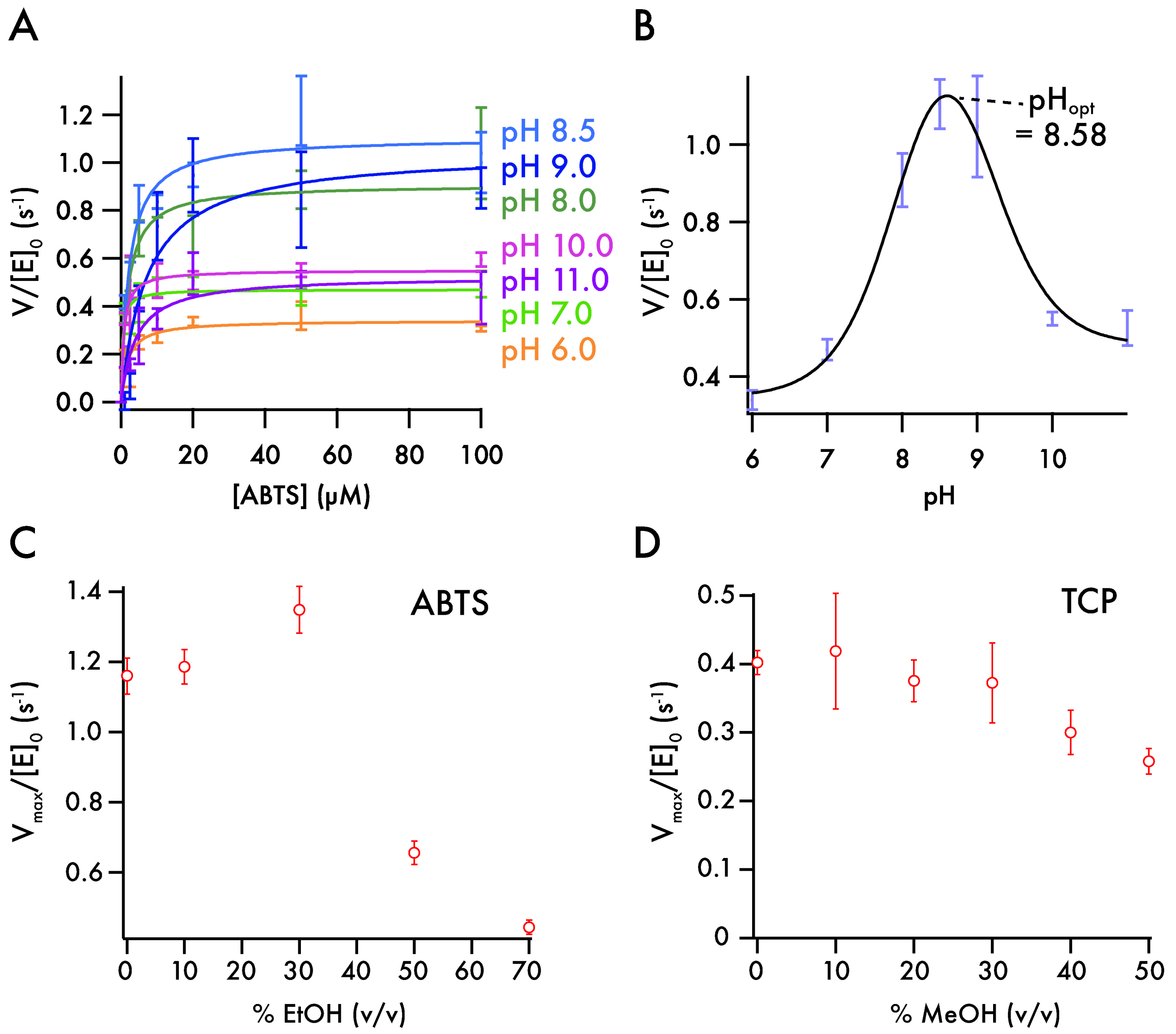


**Supplementary Figure 6. Steady-state kinetics of ABTS oxidation at variable pH and in the presence of organic solvents**

**A.** Michaelis-Menten kinetics for the oxidation of ABTS by C45 and $H_2O_2$ at pH 6.0, 7.0, 8.0, 8.5, 9.0, 10.0 and 11.0. C45, ABTS and $H_2O_2$ were mixed to give final concentrations of 0.25 µM, 0 - 100 µM and 100 µM respectively. Kinetics were measured at 25 °C in 100 mM KCl and 20 mM buffer (potassium phosphate for pH 6.0 - 8.0; CHES for pH 8.5 - 9.0; bis-tris for pH 10.0 - 11.0). Michaelis-Menten parameters for the data contained in **A** are presented in Supplementary Fig. Table 1. **B.** $k_{cat}$ plotted as a function of pH. Data were fit to a double sigmoidal model indicating p$K_a$'s at 8.0 and 9.1, and a pH optimum for activity at pH 8.58. **C.** The oxidation of ABTS by C45/$H_2O_2$ in increasing concentrations of ethanol. Steady state-kinetic assays were recorded in triplicate at 0, 10, 30, 50, 70 % ethanol (v/v), fit to a standard Michaelis-Menten model and the observed $V_{max}/[E]_0$ were plotted versus volume fraction of ethanol. For the aqueous component of the assays, the buffer conditions were 0.25 µM C45, 100 µM $H_2O_2$ 100 mM KCl, 20 mM CHES, pH 8.58. **D.** The oxidative dehalogenation of 2,4,6-trichlorophenol by C45/$H_2O_2$ in increasing concentrations of methanol. Steady state-kinetic assays were recorded in triplicate at 0, 10, 20, 30, 40 and 50 % methanol (v/v), fit to a standard Michaelis-Menten model and the resulting observed $V_{max}/[E]_0$ were plotted versus volume fraction of ethanol. For the aqueous component of the assays, the buffer conditions were 0.25 µM C45, 100 µM $H_2O_2$ 100 mM KCl, 20 mM CHES, pH 8.58. All data were recorded in triplicate and error bars represent the standard deviation.

**A**

$$\nu = [E]_0 k_{cat} \frac{\left(\frac{[A]_0}{K_{m(A)}} \frac{[B]_0}{K_{m(B)}}\right)}{\frac{[A]_0}{K_{m(A)}} + \frac{[B]_0}{K_{m(B)}} + \left(\frac{[A]_0}{K_{m(A)}} \frac{[B]_0}{K_{m(B)}}\right)}$$

**B**

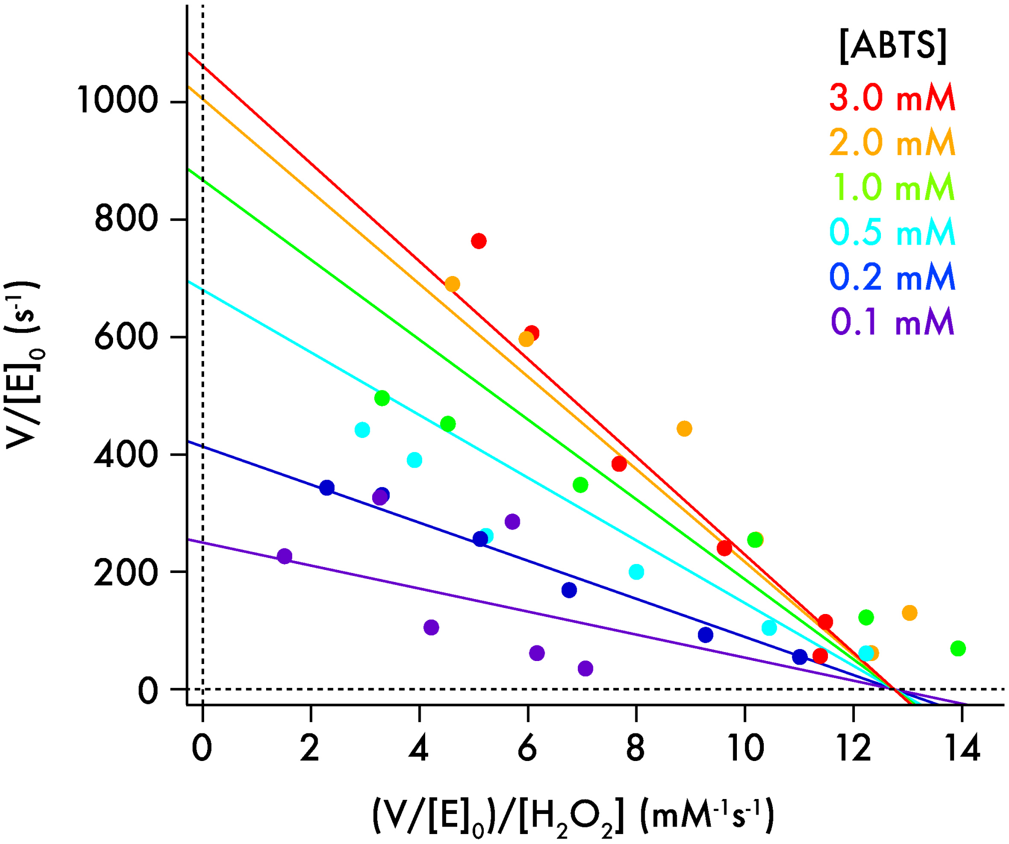


**Supplementary Figure 7. Mechanistic analysis of ABTS oxidation kinetics**
**A.** Equation describing the ping-pong steady-state kinetics exhibited by C45 for the oxidation of ABTS. **B.** Diagnostic Eadie-Hofstee plot for the oxidation of ABTS by C45. Data were collected as described in **Fig. 2**, and are fit here to the equation for ping-pong steady state kinetics.

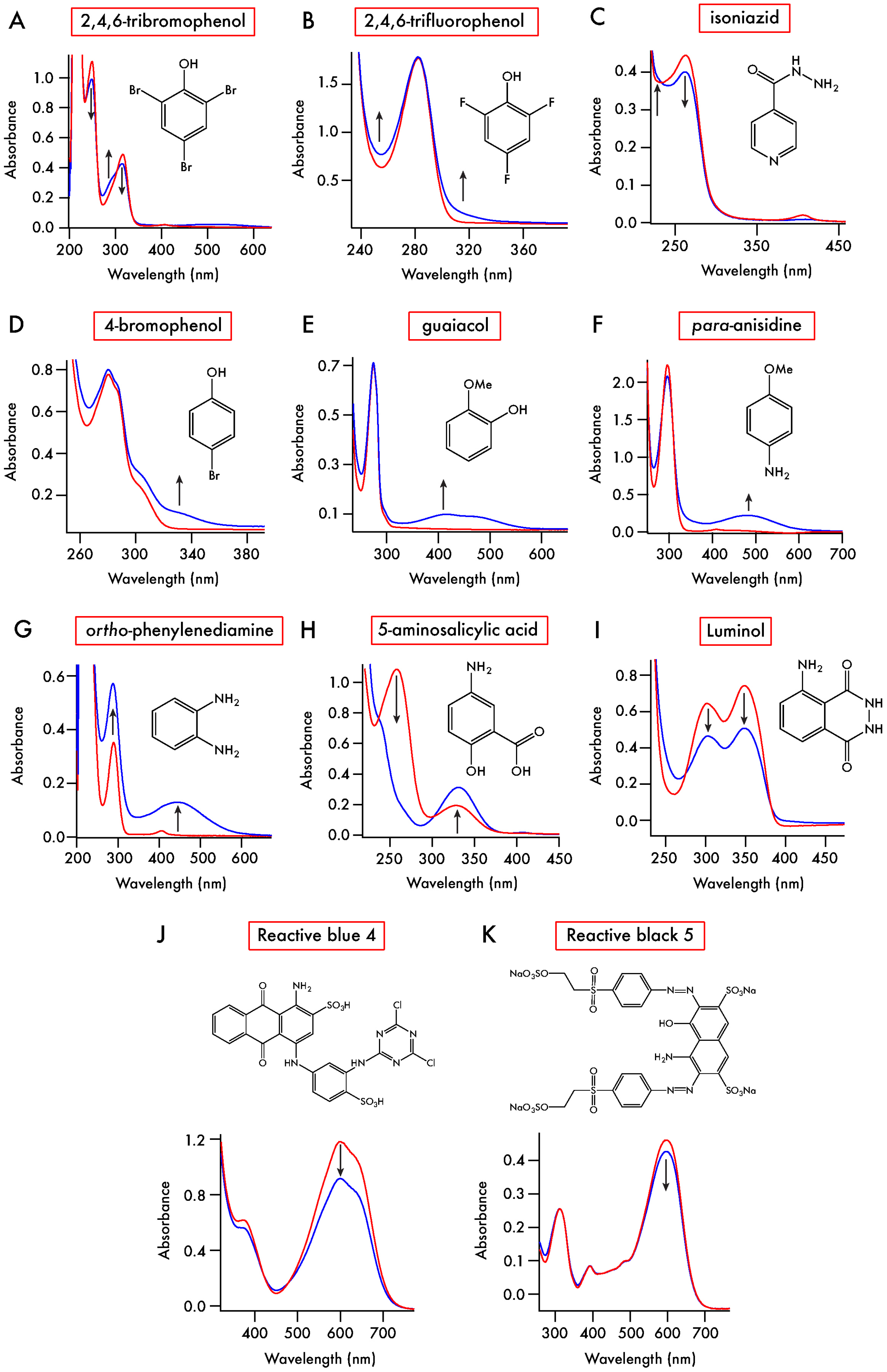


**Supplementary Figure 8. Substrate promiscuity of C45**

Representative UV/visible spectral changes on mixing 0.25 µM C45 and 100 µM $H_2O_2$ with various small molecule substrates in 100 mM KCl, 20 mM CHES, pH 8.58. Red traces are the small molecule spectra prior to the addition of C45. Arrows indicate the direction of absorbance changes on addition of C45.

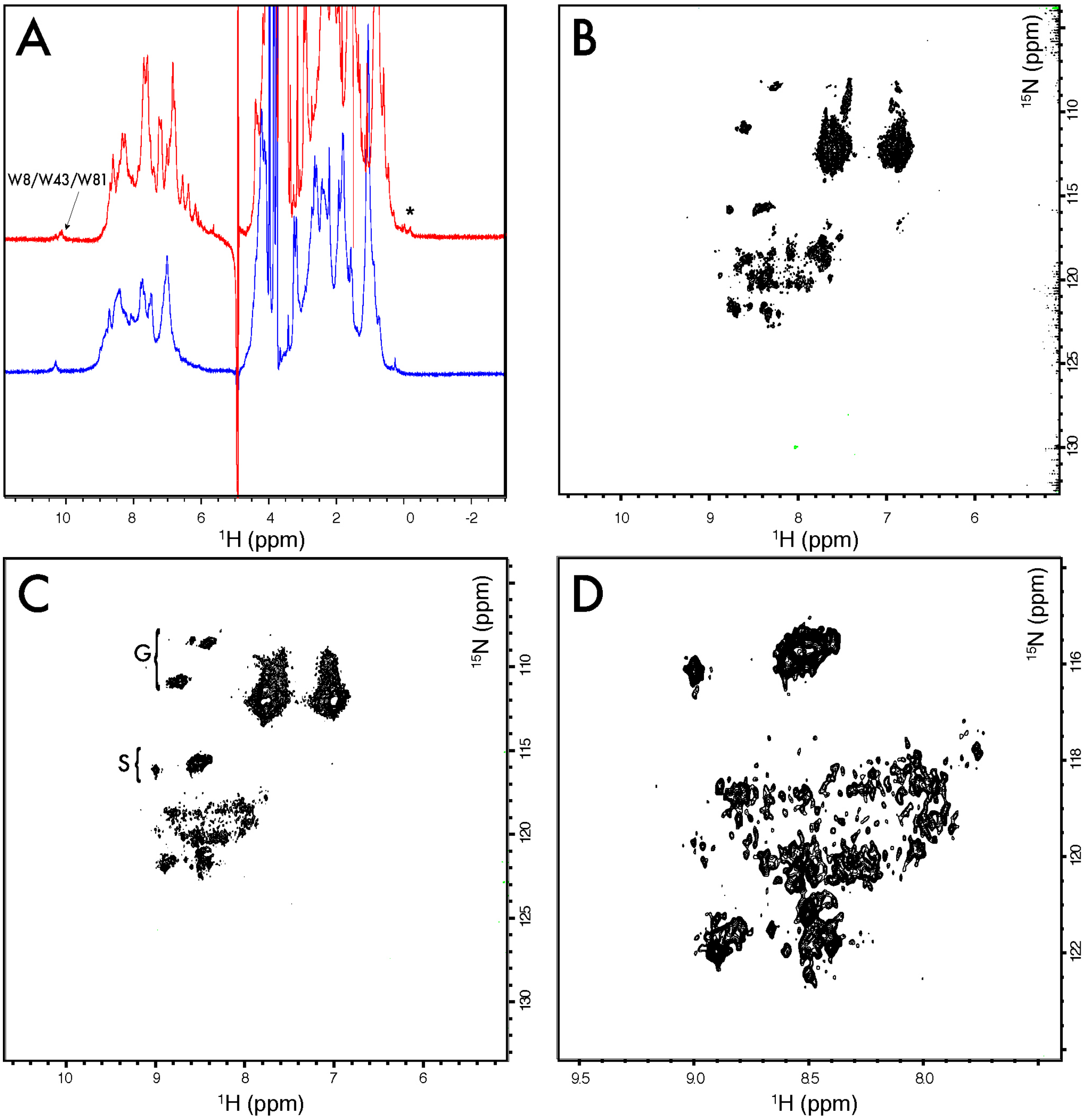


**Supplementary Figure 9. Magnetic resonance spectroscopy of C45. A-D**
$^1$H NMR and $^1$H-$^{15}$N SOFAST HMQC NMR spectra of ferric and demetallated C45. **A.** Comparison of 1D $^1$H NMR spectra recorded at 700 MHz on a 1.7 mm micro-cryocoil NMR spectrometer. The top trace shows C45 with (blue) and without (red) $Fe^{3+}$ present. In the $Fe^{3+}$ free 1D NMR spectrum the peaks appear slightly sharper and three indole NH peaks are clearly resolved (corresponding to W8, W43 and W81). In addition several up-field shifted methyl peaks are clearly visible indicative of structuring. In the $Fe^{3+}$ coordinated C45, only a single tryptophan resonance is visible which may be tentatively assigned to W8 or W81 or over-lapped peaks for both as they are separated from the paramagnetic broadening effects of the iron. **B, C** $^1$H-$^{15}$N SOFAST HMQC spectra of C45 at 700 MHz recorded in the absence (**B**) and presence (**C**) of $Fe^{3+}$. Resonances corresponding to the random coil chemical shifts of glycine (GG and SG pairs) and serine which reside in the connecting loops have characteristic $^{15}$N chemical shifts are evident. At low contour levels in **B** and particularly in **C**, numerous low intensity resonances are visible, in line with other related systems, arising from

the dynamic conformational heterogeneity and paramagnetic broadening from the $Fe^{3+}$ in **C**. These spectra differ from the usual poorly dispersed $^{1}$H chemical shifts observed in unfolded or intrinsically disordered proteins as observed elsewhere[3,12,32]. **D.** Expansion of **C** showing the numerous dispersed weak peaks observable in $Fe^{3+}$ bound C45.

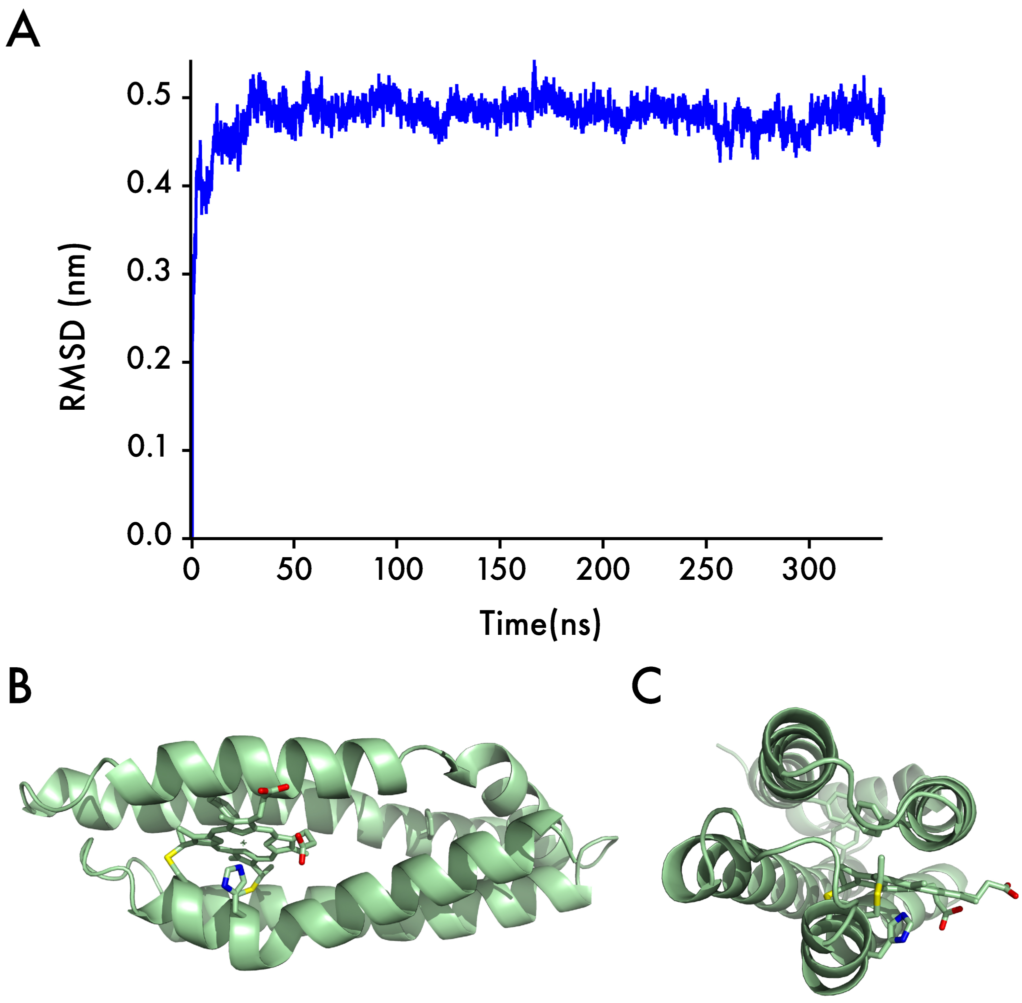


**Supplementary Figure 10. Computational modelling of C45**
**A.** Cα RMSD of C45 Molecular Dynamics simulation. This 336 ns MD simulation of C45 was run with GROMACS and the Cα RMSDs were calculated using the GROMACS tools. **B, C.** Snapshot of C45 at 336 ns from the MD simulation.

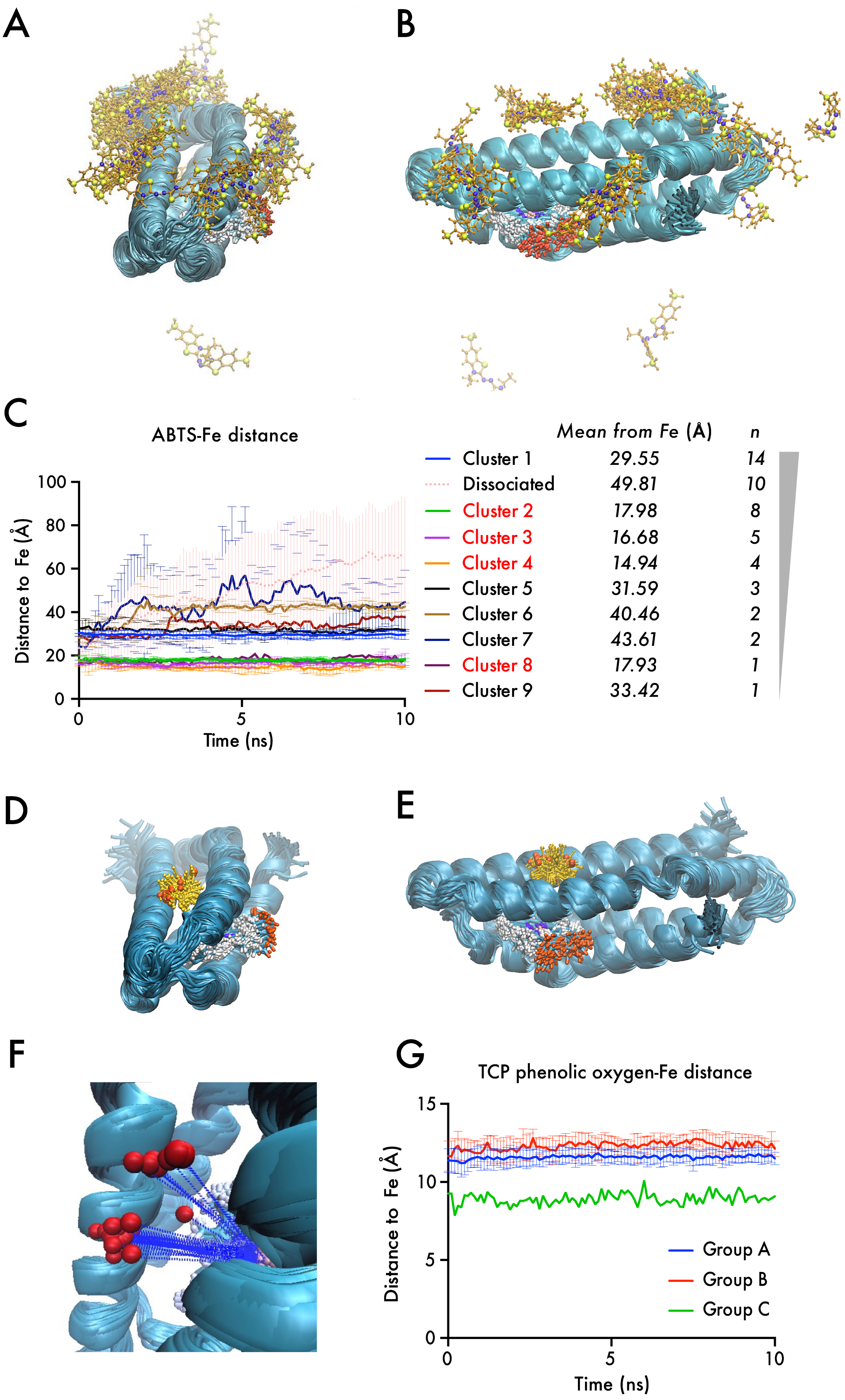


**Supplementary Figure 11. Computational analysis of substrate binding during catalysis**
**A - C.** Docking of ABTS substrate molecules to C45 using BUDE in conjunction with Molecular Dynamics simulations. **A, B.** Aligned structures of the top 50 ABTS binding positions identified by BUDE after 10 ns of simulation displayed in two orientations. ABTS carbons are displayed in yellow, heme carbons are displayed in grey. **C.** Distance between the centre of the ABTS molecules to heme iron over the course of the MD simulation, grouped by starting position. Error bars indicate the

standard deviation. The mean ABTS-Fe distance is displayed on the right of the graph, and the number of ABTS molecules in each cluster (n) is indicated. Clusters where the ABTS distance is < 20 Å are highlighted in red. **D - G.** Docking of TCP substrate molecules to C45 using BUDE in conjunction with Molecular Dynamics simulations. **D, E.** Aligned structures of the top 50 TCP binding positions identified by BUDE after 10 ns of simulation displayed in two orientations. TCP carbons are displayed in yellow, heme carbons are displayed in grey. **F.** Position of the TCP phenolic oxygen from the top 50 TCP binding positions after 10 ns Molecular Dynamics simulation. **G.** Distance between the TCP phenolic oxygen to heme iron over the course of the MD simulation, grouped by starting position. Error bars indicate the standard deviation. The number of TCP molecules (n) in each binding cluster and mean Fe-O(TCP) distances for the three clusters are: n = 30 and 11.59 Å (group A); n = 9 and 12.32 Å (group B); n = 1 and 8.91 Å (group C).

| pH | $K_{m(ABTS)}$ (µM) | $k_{cat}$ ($s^{-1}$) | $k_{cat}/K_{m(ABTS)}$ ($M^{-1}s^{-1}$) |
|---|---|---|---|
| 6.0 | 1.81 ± 0.66 | 0.34 ± 0.03 | 1.88 ± 0.71 x $10^5$ |
| 7.0 | 4.10 ± 0.23 | 0.47 ± 0.03 | 1.15 ± 0.65 x $10^5$ |
| 8.0 | 1.80 ± 0.69 | 0.91 ± 0.07 | 5.06 ± 0.20 x $10^5$ |
| 8.5 | 2.22 ± 0.61 | 1.11 ± 0.06 | 5.00 ± 0.14 x $10^5$ |
| 9.0 | 7.23 ± 3.21 | 1.05 ± 0.13 | 1.45 ± 0.67 x $10^5$ |
| 10.0 | 7.20 ± 0.16 | 0.55 ± 0.02 | 0.76 ± 0.03 x $10^4$ |
| 11.0 | 4.07 ± 1.42 | 0.53 ± 0.10 | 1.30 ± 0.52 x $10^5$ |
| **Temperature (°C)** | **$K_{m (ABTS)}$ (µM)** | **$k_{cat}$ ($s^{-1}$)** | **$k_{cat}/K_{m(ABTS)}$ ($M^{-1}s^{-1}$)** |
| 20 | 1.72 ± 0.15 | 0.61 ± 0.00 | 3.55 ± 0.31 x $10^5$ |
| 30 | 2.25 ± 0.01 | 0.94 ± 0.12 | 4.18 ± 0.53 x $10^5$ |
| 40 | 4.55 ± 0.72 | 1.62 ± 0.02 | 3.56 ± 0.57 x $10^5$ |
| 50 | 5.57 ± 1.22 | 2.04 ± 0.16 | 3.66 ± 0.85 x $10^5$ |
| 60 | 5.26 ± 0.58 | 2.38 ± 0.07 | 4.53 ± 0.52 x $10^5$ |
| 70 | 11.33 ± 1.75 | 2.97 ± 0.14 | 2.62 ± 0.42 x $10^5$ |

**Supplementary Table 1. Michaelis-Menten parameters for the pH and temperature dependence of ABTS oxidation by C45 and $H_2O_2$ under limiting [$H_2O_2$]**
All Data were recorded in triplicate and fit to a standard Michaelis-Menten model. For the dataset recorded with varying pH, the buffer conditions are described in Supplementary Fig. 6. For the dataset recorded with varying temperature, reactions were carried out in the following conditions: 0.25 µM C45, 100 µM $H_2O_2$, 100 mM KCl, 20 mM CHES, pH 8.58 with [ABTS] varied between 0-100 µM.
